# Thyroid Stimulating Hormone Receptor (TSHR) Intron 1 Variants Are Major Risk Factors for Graves' Disease in Three European Caucasian Cohorts

**DOI:** 10.1371/journal.pone.0015512

**Published:** 2010-11-25

**Authors:** Rafał Płoski, Oliver J. Brand, Beata Jurecka-Lubieniecka, Maria Franaszczyk, Dorota Kula, Paweł Krajewski, Muhammad A. Karamat, Matthew J. Simmonds, Jayne A. Franklyn, Stephen C. L. Gough, Barbara Jarząb, Tomasz Bednarczuk

**Affiliations:** 1 Department of Medical Genetics, Medical University of Warsaw, Warsaw, Poland; 2 Department of Forensic Medicine, Medical University of Warsaw, Warsaw, Poland; 3 Centre for Endocrinology, Diabetes and Metabolism, University of Birmingham, Birmingham, United Kingdom; 4 Oxford Centre for Diabetes, Endocrinology and Metabolism, University of Oxford, Oxford, United Kingdom; 5 Department of Nuclear Medicine and Endocrine Oncology, Maria Sklodowska-Curie Memorial Cancer Center and Institute of Oncology, Gliwice, Poland; 6 Department of Endocrinology, Medical University of Warsaw, Warsaw, Poland; Ohio State University Medical Center, United States of America

## Abstract

**Background:**

The thyroid stimulating hormone receptor (TSHR) gene is an established susceptibility locus for Graves' disease (GD), with recent studies refining association to two single nucleotide polymorphisms (SNPs), rs179247 and rs12101255, within *TSHR* intron 1.

**Methodology and Principal Findings:**

We aimed to validate association of rs179247 and rs12101255 in Polish and UK Caucasian GD case-control subjects, determine the mode of inheritance and to see if association correlates with specific GD clinical manifestations. We investigated three case-control populations; 558 GD patients and 520 controls from Warsaw, Poland, 196 GD patients and 198 controls from Gliwice, Poland and 2504 GD patients from the UK National collection and 2784 controls from the 1958 British Birth cohort. Both rs179247 (P = 1.2×10^−2^–6.2×10^−15^, OR = 1.38–1.45) and rs12101255 (P = 1.0×10^−4^–3.68×10^−21^, OR = 1.47–1.87) exhibited strong association with GD in all three cohorts. Logistic regression suggested association of rs179247 is secondary to rs12101255 in all cohorts. Inheritance modeling suggested a co-dominant mode of inheritance in all cohorts. Genotype-phenotype correlations provided no clear evidence of association with any specific clinical characteristics.

**Conclusions:**

We have validated association of *TSHR* intron 1 SNPs with GD in three independent European cohorts and have demonstrated that the aetiological variant within the *TSHR* is likely to be in strong linkage disequilibrium with rs12101255. Fine mapping is now required to determine the exact location of the aetiological DNA variants within the *TSHR*.

## Introduction

The thyroid stimulating hormone receptor (TSHR) is primarily expressed on the thyroid follicular cell surface membrane and via its ligand, TSH, is one of the key regulators of thyroid growth and hormone production. TSHR autoantibodies with either stimulatory (TSAb) or blocking (TBAb) activity are a key feature of autoimmune thyroid disease (AITD), with TSAb having a pre-dominant effect in Graves' disease (GD) leading to hyperthyroidism [1]. Genetic variation within the *TSHR* region may influence TSHR structure, expression and/or post-translational processing, which in turn could initiate or exacerbate the autoimmune response against the TSHR in GD.

Original *TSHR* genetic studies focused on several non-synonymous single nucleotide polymorphisms (nsSNPs) and despite a number of studies, no replicable GD associations emerged [2]–[8]. Genome wide linkage analysis subsequently suggested a GD locus in this chromosome region [9]. Later association screening by two independent studies identified strong association within *TSHR* intron 7 in Japanese and *TSHR* intron 1 in UK Caucasian cohorts, which provided the first convincing evidence for *TSHR* association with GD [10], [11]. The Wellcome Trust Case Control Consortium (WTCCC) identified association of a further *TSHR* nsSNP rs3783941 (Arg248Ser) with GD in a genome wide screen of 15,000 nsSNPs in 900 UK Caucasian GD cases and 1500 controls [12]. Recent detailed association mapping of 98 SNPs across 800Kb of the *TSHR* region, refined association to within 40Kb of *TSHR* intron 1 in 768 GD patients and 768 controls of UK Caucasian origin [13]. Strongest SNP associations were rs179247 (P = 8.9×10^−8^, OR = 1.53) and rs12101255 (P = 1.9×10^−7^, OR = 1.55), with logistic regression suggesting these SNPs or variants in strong linkage disequilibrium (LD) may drive the association signal in the region [13]. Furthermore, rs179247 and rs12101255 were associated with reduced expression of full length TSHR mRNA relative to two truncated splice variants in thyroid tissue [13]. The aim of the current study was to determine whether rs179247 and rs12101255 were also associated with GD in two Polish case-control collections. Our secondary aim was to further investigate association in an extended UK cohort, combined with detailed inheritance and logistic regression analysis to attempt to split association between these two SNPs. Finally, sub-phenotype analysis was performed to look for association with specific GD clinical characteristics.

## Methods

All GD patients in the cohorts investigated in the current study were unrelated and gave informed written consent and the project was approved by the relevant local research committees, including; the South Birmingham Research Ethics committee (MRec/01/7/60), Medical Research Centre, Polish academy of science ethics committee and the Maria Sklodowska-Curie Memorial Cancer Centre and Institute of Oncology, Gliwice Branch ethics committee.

### Subjects

Two independent Polish GD case-control collections were investigated in the current study. The first, from Warsaw, included 558 Caucasian GD patients recruited from the Department of Endocrinology, Medical University of Warsaw, Poland. The second was from Gliwice, and consisted of 196 Caucasian GD patients, ascertained at the Department of Nuclear Medicine and Endocrine Oncology, Centre of Oncology, Gliwice, Poland. Criteria for clinical diagnosis were the same for both collections, which were based on clinical and biochemical evidence of hyperthyroidism, confirmed by the presence of diffuse goiter, detectable TSHR autoantibodies (TRAK Lumitest, B.R.A.H.M.S Diagnostica GmbH, Germany) and/or increased radioiodine uptake, as described previously [14]. The severity of ophthalmopathy was assessed according to the NOSPECS classification, with patients ≥ NOSPECS class III considered clinically evident. Independent, geographically matched control groups were recruited for each of the two cohorts (Warsaw and Gliwice). The Warsaw control group contained 520 anonymous unrelated Polish adults selected from a national repository, in-part established for case-control studies [15]. The Gliwice control group consists of 198 healthy subjects with no family history of GD or other autoimmune disorder, as described previously [16]. All control groups were established for case-control association studies and have been widely investigated and characterised previously [15], [16].

The UK cohort consisted of 2504 GD patients which make up part of the AITD UK national collection. Of these, 308 GD patients were investigated previously [13] with an additional 2196 GD patients from the UK national collection also investigated [17]. In addition, 2784 geographically matched controls were obtained from the 1958 British Birth Cohort (1958 BBC) [18]. The clinical characteristics of both the Polish and UK GD National collection are displayed in Table S1.

### Genotyping

Genotyping of rs179247 and rs12101255 was performed using TaqMan® SNP genotyping technology (Applied Biosystems, Foster City, USA) according to the manufacturer's protocol, independently in the three respective centres.

### Statistical analyses

Prospective power calculations in both Polish and UK cohorts demonstrated we had >99% power (α = 0.05) to detect a significant difference in allele frequencies between cases and controls, assuming an OR = 1.50 as reported previously [13]. To ensure genotyping accuracy Hardy-Weinberg equilibrium (HWE) was calculated for all cohorts, with P<0.05 considered indicative of deviation from HWE. Allele and genotype association tests assuming recessive, dominance and co-dominance were compared between GD cases and controls using the Chi-squared test of significance within the Statistica (StatSoft Inc., Tulsa, USA) statistical package. LD between the two SNPs was measured using the pairwise LD measures D' and r^2^ and LD blocks were subsequently defined using the Gabriel *et al* algorithm [19]. Briefly, this algorithm calculates 95% confidence intervals (CI) of D' for each pair of loci and subsequently groups sets of markers into either; “strong LD” or “strong recombination” based on the 95% CI [19]. Strong LD is defined if the upper 95% CI has a D'>0.98 and the lower D' CI is ≥0.7 [19]. Strong recombination is defined when the D' upper 95% CI is D'<0.9. Each LD block is defined when 95% or more of the region is classified as pairwise “strong LD”. The algorithm allows for 5% of an LD block to show some evidence of recombination [19]. Based on this LD block definition haplotype counts of GD cases and controls were constructed and analysed for association within the computer program Haploview version 4.2, which employs an expectation maximization algorithm (http://www.broad.mit.edu/mpg/haploview) [20]. Logistic regression analysis was performed using the SPSS statistical package (SPSS, UK) within the Polish cohorts and the PLINK statistical package was used in the UK [21].

### Clinical Phenotypes

Association analysis of rs179247 and rs12101255 with specific clinical manifestations of GD were performed in all 3 cohorts for; age of GD diagnosis (≤30 years *vs* ≥31 years), severity of ophthalmopathy based on NOSPECS classification (NOSPECS 0 – 1 *vs* NOSPECS 2 – 6) and smoking status (smokers *vs* non-smokers).

## Results

### Allele and genotype distribution

GD cases and controls in all 3 cohorts were within HWE (P>0.05). In the Warsaw and Gliwice cohorts allele A of rs179247 was more frequent in GD patients (P = 6.0×10^−5^–9.0×10^−3^ and OR = 1.42–1.45). Similarly, the T allele of rs12101255 displayed an increased frequency in both Warsaw and Gliwice GD patients compared to controls (P = 3.0×10^−5^–4.0×10^−5^, OR = 1.47–1.87, respectively) (Table 1). As there was no significant difference in allele frequencies of rs179247 and rs12101255 between the Warsaw and Gliwice cohorts (P>0.05) we combined the two Polish cohorts (GD = 754, controls = 718), further confirming strong association for rs179247 (P = 3.00×10^−6^, OR = 1.43 [95% CI = 1.23–1.65]) and rs12101255 (P = 1.00×10^−7^, OR = 1.57 [95% CI = 1.34–1.84]) (Table 1).

**Table 1:** TSHR genotype and allele frequencies in Warsaw, Gliwice, combined Polish cohorts and UK case-control cohorts.

*TSHR* SNP	Cohort	Alleles/Genotypes	Controls (%)	GD (%)	P	OR	95% CI
**rs179247:**	**Warsaw**	AA	81 (15.6)	139 (24.9)	2.0×10^−4^	1.42	1.20–1.68
		AG	259 (49.0)	270 (48.8)			
		GG	180 (34.6)	149 (26.7)			
		A	421 (40.5)	548 (49.1)	6.0×10^−5^		
		G	619 (59.5)	568 (50.9)			
	**Gliwice**	AA	34 (17.1)	58 (29.6)	1.2×10^−2^	1.45	1.10–1.93
		AG	98 (49.2)	84 (42.9)			
		GG	67 (33.7)	54 (27.6)			
		A	166 (41.7)	200 (51.0)	9.0×10^−3^		
		G	232 (58.3)	192 (49.0)			
	**Polish Cohorts Pooled**	AA	115 (16.0)	197 (26.1)	3.0×10^−6^	1.43	1.23–1.65
		AG	356 (49.7)	354 (46.9)			
		GG	247 (34.4)	203 (26.9)			
		A	586 (40.8)	748 (49.6)	2.0×10^−6^		
		G	850 (59.2)	760 (50.4)			
	**UK GD National Collection**	AA	737 (29.0)	879 (37.6)	8.6×10^−14^	1.38	1.27–1.49
		AG	1243 (48.9)	1110 (47.4)			
		GG	561 (22.1)	351 (15.0)			
		A	2717 (53.5)	2868 (61.3)	6.2×10^−15^		
		G	2365 (46.5)	1812 (38.7)			
**rs12101255:**	**Warsaw**	TT	35 (6.7)	75 (13.4)	1.0×10^−4^	1.47	1.23–1.77
		TC	212 (40.8)	245 (43.9)			
		CC	273 (52.5)	238 (42.7)			
		T	282 (27.1)	395 (35.4)	3.0×10^−5^		
		C	758 (72.9)	721 (64.6)			
	**Gliwice**	TT	17 (8.6)	32 (16.3)	2.0×10^−4^	1.87	1.39–2.53
		TC	71 (35.9)	94 (48.0)			
		CC	110 (55.6)	70 (35.7)			
		T	105 (26.5)	158 (40.3)	4.0×10^−5^		
		C	291 (73.5)	234 (59.7)			
	**Polish Cohorts Pooled**	TT	52 (7.2)	107 (14.2)	1.0×10^−7^	1.57	1.34–1.84
		TC	283 (39.4)	339 (45.0)			
		CC	383 (53.3)	308 (40.8)			
		T	387 (27.0)	553 (36.7)	2.0×10^−8^		
		C	1049 (73.1)	955 (63.3)			
	**UK GD National Collection**	TT	338 (13.4)	482 (20.9)	4.14×10^−20^	1.49	1.37–1.61
		TC	1148 (45.3)	1136 (49.3)			
		CC	1046 (41.3)	687 (29.8)			
		T	1824 (36.0)	2100 (45.6)	3.68×10^−21^		
		C	3240 (64.0)	2510 (54.4)			

Both SNPs are displayed 5′ – 3′ on the positive DNA strand. P-values generated via chi-squared (χ^2^) tests for both alleles (2×2) and genotypes (co-dominant) are displayed. The odds ratios (ORs) and 95% CI have been calculated from the minor allele in each SNP.

To further investigate association and interaction between the two markers, we investigated rs179247 and rs12101255 in a GD data set from the UK national collection of AITD patients. Both SNPs demonstrated strong association in the UK data set with rs179247 allele A (P = 8.60×10^−14^, OR = 1.38 [95% CI = 1.27–1.49]) and rs12101255 allele T (P = 4.14×10^−20^, OR = 1.49 [95% CI = 1.37–1.61]). To determine whether the strong associations could be explained by either a dominant or recessive effect, we modeled the mode of inheritance for these variants and found that neither, dominant or recessive models improved association, suggesting a co-dominant effect within the region (Table S2).

### Haplotypes

The two SNPs demonstrated strong but incomplete LD (D' = 0.98 and r^2^ = 0.50), with three common (minor allele frequency (MAF) ≥0.11) haplotypes observed (Table 2). The strongly associated alleles at each individual SNP locus, rs179247 (A) and rs12101255 (T) form a single haplotype (haplotype number 1) demonstrating the strongest association with GD in all populations (Combined Polish P = 2.0×10^−8^, UK cohort P = 3.2×10^−21^). Interestingly, if the associated allele at rs12101255 was changed to the non-associated rs1210255 (C) and rs179247 (A) remained the same (haplotype 2) there was no association in any population (Combined Polish P = 0.57, UK cohort P = 0.05), suggesting rs12101255 (A) may drive the association within haplotype 1. The non-associated alleles of rs179247 (G) – rs12101255 (C) make up haplotype number 3 and display an increased frequency in controls compared to GD patients.

**Table 2:** Haplotype association tests between TSHR rs179247 and rs12101255 SNPs.

Cohorts	*TSHR* SNP	Allele	Haplotype Number	Controls Frequency	GD Frequency	P	OR	95% CI
**Warsaw**	rs179247	A	1	26.9	35.1	5.0×10^−5^	1.45	1.21–175
	rs12101255	T						
**Gliwice**	rs179247	A	1	26.3	40.2	4.0×10^−5^	1.84	1.36–2.49
	rs12101255	T						
**Polish Cohorts Pooled**	rs179247	A	1	26.8	36.4	2.0×10^−8^	1.55	1.32–1.81
	rs12101255	T						
**UK GD National Collection**	rs179247	A	1	36.0	45.0	3.2×10^−21^	1.47	1.36–1.59
	rs12101255	T						
**Warsaw**	rs179247	A	2	13.7	14.3	0.67	-	-
	rs12101255	C						
**Gliwice**	rs179247	A	2	15.4	11.0	0.07	-	-
	rs12101255	C						
**Polish Cohorts Pooled**	rs179247	A	2	14.2	13.5	0.57	-	-
	rs12101255	C						
**UK GD National Collection**	rs179247	A	2	17.0	16.0	0.05	-	-
	rs12101255	C						
**Warsaw**	rs179247	G	3	59.4	50.6	4.0×10^−4^	0.70	0.59–0.83
	rs12101255	C						
**Gliwice**	rs179247	G	3	58.2	48.9	8.0×10^−3^	0.70	0.53–0.92
	rs12101255	C						
**Polish Cohorts Pooled**	rs179247	G	3	59.1	50.2	1.0×10^−6^	0.70	0.60–0.81
	rs12101255	C						
**UK GD National Collection**	rs179247	G	3	46.0	39.0	1.0×10^−15^	0.72	0.67–078
	rs12101255	C						

The frequency of the 3 haplotypes between rs179247 and rs12101255 in GD cases and controls in 3 independent cohorts and combined Polish cohort are displayed. The P-value of the Chi-squared (χ^2^) test is displayed, with respective Odds ratios (OR) and 95% CI for haplotypes showing evidence for association (P<0.05) with GD.

### Logistic Regression Analysis

Since haplotype analysis suggested an important role for rs12101255 in driving association, we performed logistic regression to try and split association between the two SNPs. In both the combined Polish data set and in the UK national collection both SNPs demonstrated strong association when entered into the model (co-dominant), as expected. However, after conditioning on each SNP, rs12101255 remained associated after conditioning for rs179247 (Polish P = 2.0×10^−3^, UK P = 4.23×10^−8^), whereas rs179247 became non-associated after conditioning for rs12101255 (Polish P = 0.36, UK P = 0.16). This suggests association at rs179247 maybe driven by rs12101255 or that rs12101255 is in stronger LD with the aetiological variants within the region.

### Genotype phenotype correlations

We looked for replicable clinical phenotype associations with the two SNPs across all 3 cohorts. The non-associated G allele of rs179247 displayed a slight increase in frequency in smokers compared to non-smokers, although this association was not replicated across all 3 cohorts. No other clinical associations were detected across any of the 3 cohorts (Tables S3 and S4).

## Discussion

The present study provides convincing replication and further association mapping of *TSHR* intron 1 association with GD in European Caucasians. The minor alleles of rs179247 (A) and rs12101255 (T) were increased in GD patients compared to controls with mode of inheritance suggesting a co-dominant effect. The magnitude of genetic effects observed in the current study are similar (OR≈1.50) to those observed in the original report [13], confirming that the *TSHR* represents a major susceptibility locus for GD in-line with other previously identified non-HLA susceptibility loci [22] including *CTLA4* [23] and *PTPN22* [24]. We were unable to identify any convincing, replicable evidence for genetic association with any specific GD clinical features. An increased frequency of the non-associated G allele in smokers of the Warsaw cohort represented the only association identified, however this failed replication in all other cohorts. This maybe because GD represents a heterogeneous disorder with various GD specific clinical features, which may require even larger disease cohorts to detect such effects if they exist.

Previous Japanese investigations identified numerous SNPs within *TSHR* introns 7 and 8 strongly associated with GD [11]. However, more recent screening of an extended region of the *TSHR* (800Kb) implementing Tag SNPs in a UK Caucasian cohort, ruled out any association within introns 7–8 in Caucasians [13]. The current study replicates and furthers our understanding of *TSHR* intron 1 association in European Caucasian GD patients. Further studies are now required in Japanese and other ethnic backgrounds to independently confirm previous reports of association within *TSHR* intron 7. Indeed, it should be noted that Hiratani *et al* investigated 3 variants within intron 1 and identified some evidence of association with *TSHR* intron 1 SNP, rs2268474 (P = 0.026) in the Japanese [11]. It is particularly important that these findings are confirmed, since it could have future implications on the usefulness of exploiting different LD structures among different ethnic groups (trans-ethnic mapping), to refine genetic association.

Since rs179247 and rs12101255 are located 18.5Kb apart within *TSHR* intron 1 we attempted to resolve the issue of whether the two SNPs had independent effects or if the effect of one SNP was secondary to LD with the other. Logistic regression in all cohorts suggested that the effect of rs179247 could be secondary to rs12101255. This conclusion was consistent with single SNP analysis, which demonstrated a greater magnitude of genetic association with rs12101255 compared to rs179247 in all our cohorts. Furthermore, haplotype analysis demonstrated that the associated allele T of rs12101255 drives the highly associated haplotype of rs179247 (A) and rs12101255 (T). The increased size of the National AITD cohort of 2504 GD patients compared to the smaller dataset of 768 UK Caucasian GD investigated previously has allowed differentiation between these two highly associated SNPs [13].

In conclusion, our results confirm strong association of *TSHR* intron 1 SNPs with GD in three European Caucasian data sets and for the first time association in Polish GD subjects. Detailed investigation of all genetic variants within the region to identify the aetiological variant/s is now required, which may lead to advances towards developing TSHR targeted therapeutics.

## Supporting Information

Table S1 **Clinical Characteristics of Polish GD patients from the Warsaw and Gliwice cohorts and the UK GD National Collection.** Shows the number of patients and percentage in brackets where appropriate, possessing specific GD clinical phenotypes in Warsaw, Gliwice Polish cohorts and UK GD National Collection.
(DOC)

Table S2 **Association test results for rs179247 and rs12101255 assuming either dominant or recessive modes of inheritance.** The table displays association tests for rs179247 and rs12101255 in the GD case-control cohorts from Warsaw, Gliwice, Polish combined and UK GD national collection, assuming either dominance or recessive mode of inheritance. Allele counts are compared by means of the χ2 test of significance, with the χ2 P displayed. P<0.05 considered indicative of a significant difference between GD cases and controls.
(DOC)

Table S3 **Shows the genotypes of rs179247 with specific GD clinical features.** Each clinical feature is compared with genotype counts in the both two Polish and UK cohorts, using the following segregations; age of GD onset (<30 years old vs ≥30 years old), severity of ophthalmopathy determined by NOSPECS classification (NOSPECS <2 vs NOSPECS ≥2), smoking status (current or previous smoker vs non-smoker) and in the UK GD National Collection cohort only, thyroid antibody status whether positive for one of either thyroid peroxidise, thyroglobulin or TSHR (positive vs negative) and presence or abscence of a difuse, palpable goitre (presence vs abscence). The Chi-squared test P-value comparing genotype and allele counts is displayed. * = P<0.05.
(DOCX)

Table S4 **Shows the genotypes of rs12101255 with specific GD clinical features.** Each clinical feature is compared with genotype counts in the both two Polish and UK cohorts, using the following segregations; age of GD onset (<30 years old vs ≥30 years old), severity of ophthalmopathy determined by NOSPECS classification (NOSPECS <2 vs NOSPECS ≥2), smoking status (current or previous smoker vs non-smoker) and in the UK GD National Collection cohort only, thyroid antibody status whether positive for one of either thyroid peroxidise, thyroglobulin or TSHR (positive vs negative) and presence or abscence of a difuse, palpable goitre (presence vs abscence). The Chi-squared test P-value comparing genotype and allele counts is displayed. * = P<0.05.
(DOCX)
