# Supplementary material for: Thyroid Stimulating Hormone Receptor (TSHR) Intron 1 Variants Are Major Risk Factors for Graves' Disease in Three European Caucasian Cohorts
Source: PLoS One. 2010 Nov 25;5(11):e15512. doi: 10.1371/journal.pone.0015512 (PMC2991361; doi:10.1371/journal.pone.0015512)
Supplement: Table S1 — Clinical Characteristics of Polish GD patients from the Warsaw and Gliwice cohorts and the UK GD National Collection. Shows the number of patients and percentage in brackets where appropriate, possessing specific GD clinical phenotypes in Warsaw, Gliwice Polish cohorts and UK GD National Collection. (DOC) [file pone.0015512.s001.doc]

**Table S1**

| **Phenotype** | **Warsaw Cohort** | **Gliwice Cohort** | **UK GD National Collection** |
| --- | --- | --- | --- |
| **Female Sex (%)** | **448**  (80.3) | **161**  (82.1) | **2047** (82.8) |
| **Mean age of onset in years** | **39.6** | **44.2** | **43.1** |
| **Thyroid Associated Ohthalmopathy (%)** | **185**  (34.2) | **108**  (55.1) | **1239**  (50.1) |
| **Toabbaco smokers (%)** | **219**  (43.1) | **71**  (44.1) | **1219** (49.3) |
| **Family History of Graves' disease (%)** | **150**  (29.8) | **94**  (48.2) | **765** (30.9) |
