# Supplementary material for: Thyroid Stimulating Hormone Receptor (TSHR) Intron 1 Variants Are Major Risk Factors for Graves' Disease in Three European Caucasian Cohorts
Source: PLoS One. 2010 Nov 25;5(11):e15512. doi: 10.1371/journal.pone.0015512 (PMC2991361; doi:10.1371/journal.pone.0015512)
Supplement: Table S2 — Association test results for rs179247 and rs12101255 assuming either dominant or recessive modes of inheritance. The table displays association tests for rs179247 and rs12101255 in the GD case-control cohorts from Warsaw, Gliwice, Polish combined and UK GD national collection, assuming either dominance or recessive mode of inheritance. Allele counts are compared by means of the χ2 test of significance, with the χ2 P displayed. P<0.05 considered indicative of a significant difference between GD cases and controls. (DOC) [file pone.0015512.s002.doc]

**Table S2**

| **SNP** | **Cohort** | **Model (inheritance)** | **Alleles** | **Controls (%)** | **GD (%)** | **P** | **OR** | **95% CI** |
| --- | --- | --- | --- | --- | --- | --- | --- | --- |
| rs179247: | Warsaw | Dominant (A) | A | 340 (45.4) | 409 (54.6) | 5.0x10^-3^ | 1.45 | 1.12 - 1.88 |
|  |  |  | G | 180 (54.7) | 149 (42.3) |  |  |  |
|  |  | Recessive (A) | A | 81 (36.8) | 139 (63.2) | 1.4x10^-4^ | 1.79 | 1.32 - 2.43 |
|  |  |  | G | 439 (51.2) | 419 (48.8) |  |  |  |
|  | Gliwice | Dominant (A) | A | 132 (48.2) | 142 (51.8) | 0.19 | - | - |
|  |  |  | G | 67 (55.4) | 54 (44.6) |  |  |  |
|  |  | Recessive (A) | A | 34 (37.0) | 58 (63.0) | 3.0x10^-3^ | 2.03 | 1.25 - 3.27 |
|  |  |  | G | 165 (54.5) | 138 (45.5) |  |  |  |
|  | polish Cohorts Pooled | Dominant (A) | A | 471 (46.1) | 551 (53.9) | 2.0x10^-3^ | 1.42 | 1.14 - 1.77 |
|  |  |  | G | 247 (54.9) | 203 (45.1) |  |  |  |
|  |  | Recessive (A) | A | 115 (36.9) | 197 (63.1) | 2.1x10^-6^ | 1.85 | 1.43 - 2.39 |
|  |  |  | G | 603 (52.0) | 557 (48.0) |  |  |  |
|  | UK GD National Collection | Dominant (A) | A | 1980 (49.9) | 1989 (50.1) | 2.3x10^-10^ | 1.61 | 1.39 - 1.86 |
|  |  |  | G | 561 (61.5) | 351 (38.5) |  |  |  |
|  |  | Recessive (A) | A | 737 (45.6) | 879 (54.4) | 2.2x10^-10^ | 1.47 | 1.31 - 1.66 |
|  |  |  | G | 1804 (55.3) | 1461 (44.7) |  |  |  |
| rs12101255: | Warsaw | Dominant (T) | T | 247 (43.6) | 320 (56.4) | 1.0x10^-3^ | 1.48 | 1.17 - 1.89 |
|  |  |  | C | 273 (53.4) | 238 (46.6) |  |  |  |
|  |  | Recessive (T) | T | 35 (31.8) | 75 (68.2) | 2.9x10^-4^ | 2.13 | 1.40 - 3.25 |
|  |  |  | C | 484 (50.1) | 483 (49.9) |  |  |  |
|  | Gliwice | Dominant (T) | T | 88 (41.1) | 126 (58.9) | 7.7x10^-5^ | 2.24 | 1.49 - 3.36 |
|  |  |  | C | 110 (61.1) | 70 (38.9) |  |  |  |
|  |  | Recessive (T) | T | 17 (34.7) | 32 (65.3) | 0.02 | 2.05 | 1.10 - 3.83 |
|  |  |  | C | 181 (52.5) | 164 (47.5) |  |  |  |
|  | polish Cohorts Pooled | Dominant (T) | T | 335 (42.9) | 446 (57.1) | 1.6x10^-6^ | 1.65 | 1.35 - 2.03 |
|  |  |  | C | 383 (55.4) | 308 (44.6) |  |  |  |
|  |  | Recessive (T) | T | 52 (32.7) | 107 (67.3) | 1.8x10^-5^ | 2.11 | 1.49 - 2.99 |
|  |  |  | C | 666 (50.7) | 647 (49.3) |  |  |  |
|  | UK GD National Collection | Dominant (T) | T | 1486 (47.9) | 1618 (52.1) | 7.71x10^-17^ | 1.66 | 1.47 - 1.87 |
|  |  |  | C | 1046 (60.4) | 687 (39.6) |  |  |  |
|  |  | Recessive (T) | T | 338 (41.2) | 482 (58.8) | 2.55x10^-12^ | 1.72 | 1.47 - 2.00 |
|  |  |  | C | 2194 (54.6) | 1823 (45.4) |  |  |  |
