# Supplementary material for: Thyroid Stimulating Hormone Receptor (TSHR) Intron 1 Variants Are Major Risk Factors for Graves' Disease in Three European Caucasian Cohorts
Source: PLoS One. 2010 Nov 25;5(11):e15512. doi: 10.1371/journal.pone.0015512 (PMC2991361; doi:10.1371/journal.pone.0015512)
Supplement: Table S3 — Shows the genotypes of rs179247 with specific GD clinical features. Each clinical feature is compared with genotype counts in the both two Polish and UK cohorts, using the following segregations; age of GD onset (<30 years old vs ≥30 years old), severity of ophthalmopathy determined by NOSPECS classification (NOSPECS <2 vs NOSPECS ≥2), smoking status (current or previous smoker vs non-smoker) and in the UK GD National Collection cohort only, thyroid antibody status whether positive for one of either thyroid peroxidise, thyroglobulin or TSHR (positive vs negative) and presence or abscence of a difuse, palpable goitre (presence vs abscence). The Chi-squared test P-value comparing genotype and allele counts is displayed. * = P<0.05. (DOCX) [file pone.0015512.s003.docx]

| **Cohort** | **GD Phenotype** | **Measure** | **SNP rs179247** | | | | | | |
| --- | --- | --- | --- | --- | --- | --- | --- | --- | --- |
|  |  |  | **A/A** | **A/G** | **G/G** | **Genotype P-value** | **A** | **G** | **Allele P-value** |
| **Warsaw:** | **Age of GD onset (mean years):** | **< 30** | 51 | 74 | 37 | **0.06** | 176 | 148 | **0.02 *** |
|  |  | **≥ 30** | 82 | 184 | 106 |  | 348 | 396 |  |
|  | **Thyroid Ophthamopathy (NOSPECS):** | **< 2** | 84 | 166 | 85 | **0.67** | 334 | 336 | **0.54** |
|  |  | **≥ 2** | 55 | 104 | 64 |  | 214 | 232 |  |
|  | **Smoking:** | **Smoker** | 43 | 108 | 68 | **0.04 *** | 194 | 244 | **0.01 *** |
|  |  | **Non-Smoker** | 96 | 162 | 81 |  | 354 | 324 |  |
| **Gliwice:** | **Age of GD onset (mean years):** | **< 30** | 8 | 16 | 11 | **0.62** | 32 | 38 | **0.33** |
|  |  | **≥ 30** | 50 | 68 | 43 |  | 168 | 154 |  |
|  | **Thyroid Ophthamopathy (NOSPECS):** | **< 2** | 6 | 11 | 4 | **0.65** | 23 | 19 | **0.93** |
|  |  | **≥ 2** | 29 | 36 | 22 |  | 94 | 80 |  |
|  | **Smoking:** | **Smoker** | 30 | 40 | 27 | **0.89** | 100 | 94 | **0.84** |
|  |  | **Non-Smoker** | 28 | 44 | 27 |  | 100 | 98 |  |
| **UK GD National Collection:** | **Age of GD onset (mean years):** | **< 30** | 191 | 237 | 75 | **0.98** | 619 | 387 | **0.85** |
|  |  | **≥ 30** | 684 | 865 | 275 |  | 2233 | 1415 |  |
|  | **Thyroid Ophthamopathy (NOSPECS):** | **< 2** | 429 | 481 | 185 | **4.0x10^-3^ *** | 1339 | 851 | **0.77** |
|  |  | **≥ 2** | 425 | 588 | 155 |  | 1438 | 898 |  |
|  | **Smoking:** | **smoker** | 438 | 545 | 170 | **0.84** | 1421 | 885 | **0.60** |
|  |  | **Non-Smoker** | 306 | 402 | 126 |  | 1014 | 654 |  |

**Table S3**
